# Dose rounding of trastuzumab deruxtecan in chinese patients: cost savings and safety outcomes in real-world study

**DOI:** 10.3389/fonc.2026.1868388

**Published:** 2026-06-15

**Authors:** Haochun Tang, Ziying Li, Ziyuan Zhou, Lingzhi Ren, Chengsheng Xie, Jun Meng, Guohui Li

**Affiliations:** 1 Department of Pharmacy, National Cancer Center/National Clinical Research Center for Cancer/Cance Hospital & Shenzhen Hospital, Chinese Academy of Medical Sciences & Peking Union Medical College, Shenzhen, China; 2 School of Pharmacy, Guangdong Medical University, Dongguan, China; 3 The Affiliated Cancer Hospital, School of Medicine, Southern University of Science and Technology, Shenzhen, China

**Keywords:** adverse reactions, china, cost savings, dose rounding, trastuzumab deruxtecan

## Abstract

Trastuzumab deruxtecan is a life-saving but costly therapy for HER2-positive cancers. While dose rounding strategies have been proposed to reduce drug waste, their safety and cost-saving potential in real-world Chinese patients remain understudied. This study aimed to evaluate the cost savings of dose rounding for trastuzumab deruxtecan in China and compare the safety profiles of upward rounding, downward rounding, and no rounding groups. We conducted a retrospective real-world study of patients receiving trastuzumab deruxtecan between 2024 and 2025. Drug waste, cost savings, and dose deviations were calculated for each administration. Adverse events including nausea, vomiting, fatigue, and hematologic toxicities were compared across the three groups. Among 141 patients with 829 administrations, dose rounding down led to a cost saving of CNY 897, 840, while dose rounding up avoided 4, 368.3 mg of drug waste. Dose deviations ranged from 0.10% to 9.97%, well within the 10% acceptable limit. The incidence and severity of adverse events were comparable across groups, with no statistically significant differences observed. These findings indicate that dose rounding of trastuzumab deruxtecan to the nearest full vial, within a 10% deviation range, can significantly reduce drug waste and pharmacy costs without compromising safety. This strategy is both safe and feasible in Chinese patients and provides preliminary evidence for cautious implementation, pending validation in larger studies.

## 1 Introduction

In 2024, China’s total healthcare expenditure approached CNY 10 trillion, with both medical insurance funds and out-of-pocket spending increasing simultaneously (1). Drug costs represent one of the primary drivers of overall healthcare cost growth, particularly in oncology. The rapid advancement of biologics (e.g., large−molecule monoclonal antibodies) has increased medication expenses for patients with cancer, intensifying the financial burden on both healthcare systems and individuals. The global oncology drug market is projected to reach $612.7 billion by 2035 (2). This rapid cost escalation has prompted international healthcare systems to implement various measures to control cancer−related expenditures, including the use of multi−dose vials and dose rounding (3). Dose rounding involves adjusting prescription doses to the nearest full vial to minimize the use of partial vials (4). According to recommendations from the *Global initiative to establish and implement dose rounding policy for expensive cancer therapy* and *Dose Rounding of Biologic and Cytotoxic Anticancer Agents: A Position Statement of the Hematology/Oncology Pharmacy Association* endorsed by the National Comprehensive Cancer Network (NCCN) (4) (5), dose rounding within 10% of the prescribed dose does not compromise patient safety or efficacy. This practice is widely advocated internationally to reduce drug costs and avoid wastage. The therapeutic doses of most anticancer agents are calculated based on body surface area (BSA) or body weight (6). For high−volume or high−cost medications, residual quantities from partial vials constitute substantial drug wastage, which contributes to the overall cost of cancer treatment. Therefore, defining an appropriate adjustable dose range within anticancer treatment cycles is critical for cost reduction. Overseas studies have consistently demonstrated that dose rounding strategies for anticancer drugs effectively reduce medication costs (7) (8) (9), whereas relevant research in China remains limited (10).

This retrospective study evaluated the cost saving impact of dose rounding for trastuzumab deruxtecan between 2024 and 2025 at a cancer hospital. We compared the incidence and severity of adverse events among patients receiving different dose rounding strategies and assessed the clinical safety of this approach in the Chinese population. The study aims to provide evidence to support cost reduction and waste minimization of anticancer drugs in clinical practice and health policy in China. Alternative strategies, such as fixed dosing within weight bands, may further simplify administration and reduce waste, warranting future investigation.

## 2 Materials and methods

### 2.1 Study drugs

This study used trastuzumab deruxtecan for injection (Manufacturer: Baxter Oncology Gmbh; formulation: 100 mg/vial; batch numbers: 403122 (2024), 422714 (2025); unit price: CNY 3–480 per vial), an antibody-drug conjugate targeting human epidermal growth factor receptor 2 (HER2). Based on the indication, the dose of trastuzumab deruxtecan is 6.4 or 5.4 mg/kg every 3 weeks according to actual body weight. Dose modifications may be applied during treatment according to the patient’s clinical condition. Common adverse reactions associated with its treatment include nausea, vomiting, fatigue, leukopenia, neutropenia, thrombocytopenia, and musculoskeletal pain.

### 2.2 Case source and data collection

We enrolled all patients who received at least one dose of trastuzumab deruxtecan injection at a cancer hospital between 2024 and 2025.

Inclusion Criteria:

1. Confirmed diagnosis of breast cancer, non−small cell lung cancer (NSCLC), or gastric cancer;
2. Received at least one dose of trastuzumab deruxtecan;
3. Complete laboratory test results (including complete blood count, liver function, renal function) available before and after treatment, with comprehensive medical records.

Exclusion Criteria:

1. Failure to strictly adhere to the recommended dose per the drug label (the label recommends administration every 3 weeks at an initial dose of 6.4 mg/kg for gastric cancer or 5.4 mg/kg for breast cancer or (NSCLC);
2. Incomplete laboratory test results or medical records before and after treatment;
3. Dose rounding fluctuations exceeding 10%.

General patient characteristics (age, gender, height, weight, diagnosis, department, etc.), medication details, and adverse reaction occurrences (symptoms and signs, toxicity grade, etc.) of eligible patients were reviewed and extracted from the Hospital Information System (HIS). Adverse reaction causality was assessed independently by two clinical pharmacists; any disagreement was resolved by consensus with a third reviewer. Data were entered into Microsoft Excel and verified by double-entry to ensure accuracy, authenticity, and standardization.This study was a retrospective observational study approved by the Ethics Committee of the National Cancer Center/National Clinical Research Center for Cancer/Cancer Hospital and Shenzhen Hospital (Ethics Approval No.:KYLX2022-204). Only diagnostic and treatment data were collected, and no follow-up was performed. Written informed consent was waived for all patients.

### 2.3 Dose rounding rules and calculation method

Dose rounding rules for trastuzumab deruxtecan were established according to *Dose Rounding of Biologic and Cytotoxic Anticancer Agents: A Position Statement of the Hematology/Oncology Pharmacy Association*, relevant literature, and expert advice from questionnaires.

1. If the rounded dose was within 10% of the prescribed dose, the dose was rounded to the nearest minimum package unit of the drug.
2. If the dose could not be rounded to the nearest minimum package, it was rounded to a convenient whole number.

Since the formulation of trastuzumab deruxtecan was 100 mg/vial, all rounded doses were integer multiples of 100 mg.


$$\text{Dose ronding variaton range }=\;\left|\left(\text{rounded dose}/\text{precribed dose − }1\right)\right|\;\times \;100\%$$

### 2.4 Cost savings evaluation metrics

1. Waste-cost analysis: The amount of drug discarded due to incomplete vial usage, measured in milligrams. Wasted doses were determined by comparing the prescribed dose with the rounded dose.


Wasted amount (mg) (from upward) = Dose used after dose rounding (mg)－Prescribed dose (mg)

2. Theoretical dose savings: The potential cost savings achievable by rounding the patient’s actual dose to the nearest minimum package unit dose, calculated as the amount saved.


Cost savings (CNY) (from downward rounding) = (Number of prescribed vials－Number of vials after dose rounding) × Unit price (CNY/vial)

For example: Trastuzumab deruxtecan is supplied as 100 mg/vial at a price of CNY 3, 480 per vial. If the prescribed dose calculated based on body weight is 420 mg, 5 vials of trastuzumab deruxtecan would be required. According to the dose rounding rules, the dose can be rounded down to 400 mg (with a dose rounding variation range of 4.76%), and only 4 vials would be needed. This represents a cost saving of 1 vial, equivalent to CNY 3, 480. If the prescribed dose calculated based on body weight is 380 mg, the dose can be rounded up to 400 mg according to the dose rounding rules (with a dose rounding fluctuation range of 5.26%). Without rounding up, the remaining 20 mg would be discarded. Therefore, this can be regarded as avoiding the waste of 20 mg of trastuzumab deruxtecan.

### 2.5 Adverse reaction evaluation criteria

We collected adverse reactions documented in medical records and evaluated their association with trastuzumab deruxtecan according to the relevant provisions of the Measures for the Reporting and Monitoring of Adverse Drug Reactions (11), excluding adverse reactions assessed as unlikely to be related.Adverse reactions induced by trastuzumab deruxtecan were graded for toxicity and documented according to the Common Terminology Criteria for Adverse Events (CTCAE) version 4.03 (12) developed by the National Cancer Institute (NCI). Toxicity was classified into 5 grades:

G1: mild toxicity;
G2: moderate toxicity;
G3: severe toxicity;
G4: life-threatening toxicity;
G5: toxicity-related death.

### 2.6 Data analysis

Following collection of patient information, cases were screened in accordance with the inclusion and exclusion criteria, and baseline characteristics of patients receiving the study drug were extracted. A database was constructed using Microsoft Excel, and descriptive statistics were applied to evaluate the outcome indicators. Quantitative data included height, weight, body surface area, prescribed dose, rounded dose, etc. Data were classified by qualitative analysis according to the two major indicators for cost-saving evaluation, and results were compared by quantitative methods to calculate the cost savings and the drug waste reduction. The incidence of adverse reactions was analyzed using SPSS software. Categorical data were presented as case numbers and percentages, and comparisons were conducted using the chi-square test. A P-value less than 0.05 was considered statistically significant.

## 3 Results

### 3.1 Patient baseline characteristics

A total of 159 patients (906 patient-visits) who had received at least one prior trastuzumab treatment were enrolled. After applying exclusion criteria, 141 patients (829 patient-visits) were ultimately included. Baseline patient demographics were summarized in **Table 1**. Baseline characteristics including body weight, BSA, prior lines of therapy, and concomitant medications were compared across groups to assess potential confounding. The mean body weight of the rounded-up group was 60.4 kg, compared with 53.7 kg in the non-rounded group (P = 0.0003). No patients required dose rounding up beyond the 10% limit.

**Table 1:** Baseline demographics.

Characteristics	Rounded up (n=43)	Not rounded (n=55)	Rounded down (n=43)
Median age (Range) (years)	53 (37~83)	54 (35~75)	57 (29~79)
Gender, n (%)			
Male	3 (6.98)	2 (3.64)	6 (13.95)
Female	40 (93.02)	53 (96.36)	37 (86.05)
Type of malignancy, n (%)			
Breast cancer	39 (90.70)	52 (94.55)	34 (79.07)
Gastric cancer	1 (2.33)	0	3 (6.98)
Non-small cell lung cancer (NSCLC)	3 (6.98)	3 (5.45)	6 (13.95)
Karnofsky performance status, n (%)			
90–100 points	31 (72.09)	37 (67.27)	29 (67.44)
≤80 points	12 (27.91)	18 (32.73)	14 (32.56)

### 3.2 Dose rounding fluctuations

**Figure 1:**
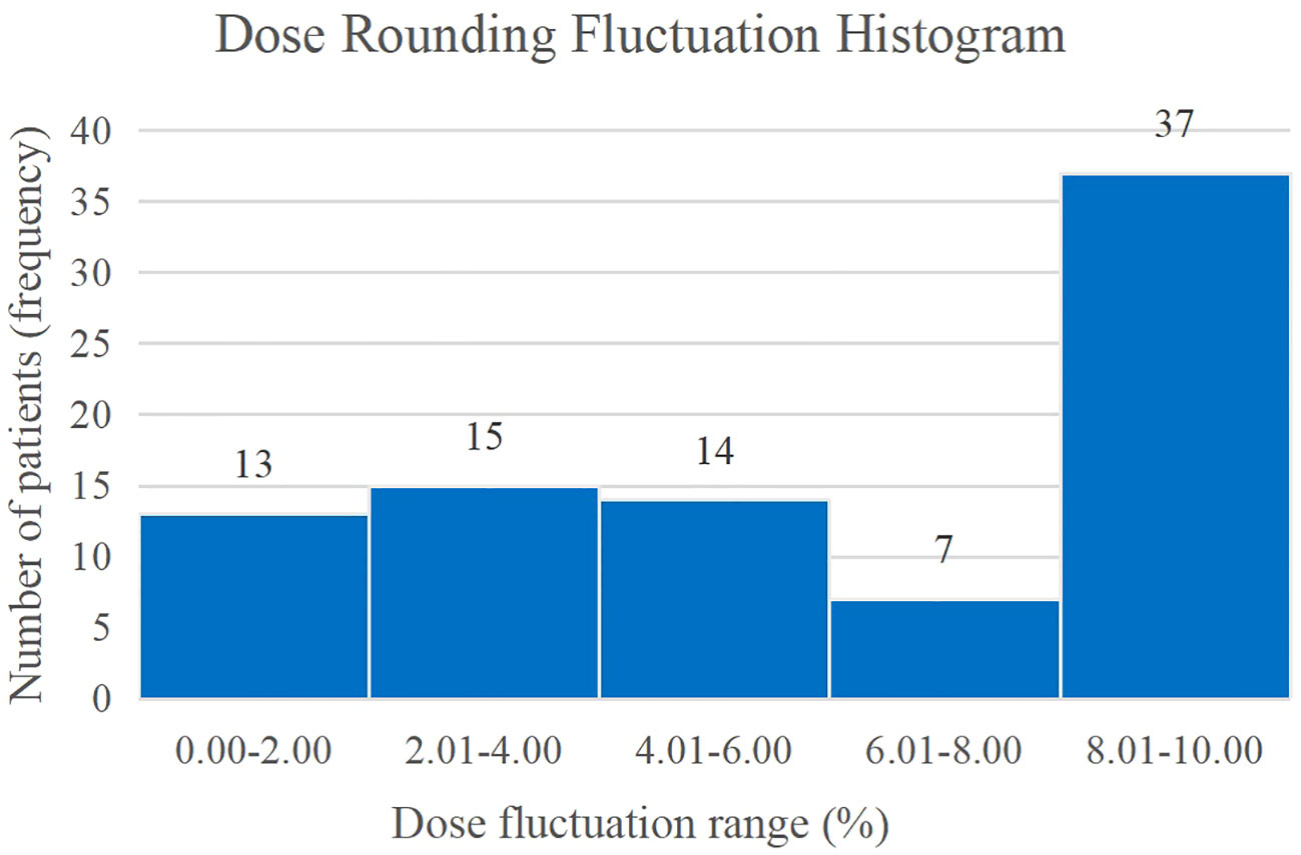
Dose rounding fluctuation histogram.

The fluctuation range of dose rounding in this study was 0.10%-9.97%. Dose rounding fluctuations for all patients were controlled within 10%, and were primarily concentrated in the range of 8.01%-10.00%, as shown in **Figure 1**.

### 3.3 Total cost savings from trastuzumab deruxtecan dose rounding

Compared with the dose of trastuzumab deruxtecan based on actual body weight, 43 patients had their doses rounded down and 43 patients had their doses rounded up. Dose rounding down led to estimated wholesale drug cost savings of CNY 897, 840, while dose rounding up prevented 4, 368.3 mg of drug waste. Detailed data are shown in **Table 2**.

**Table 2:** Cost savings from dose rounding.

Dose type	No. of patients (cases)	No. of doses administered (times)	Waste avoidance (mg)	Cost savings (CNY)
Rounded up	43	256	4368.3	-–
Not rounded	55	315	-–	-–
Rounded down	43	258	-–	897840

### 3.4 Adverse drug reaction (ADR) incidence and comparison

**Figure 2:**
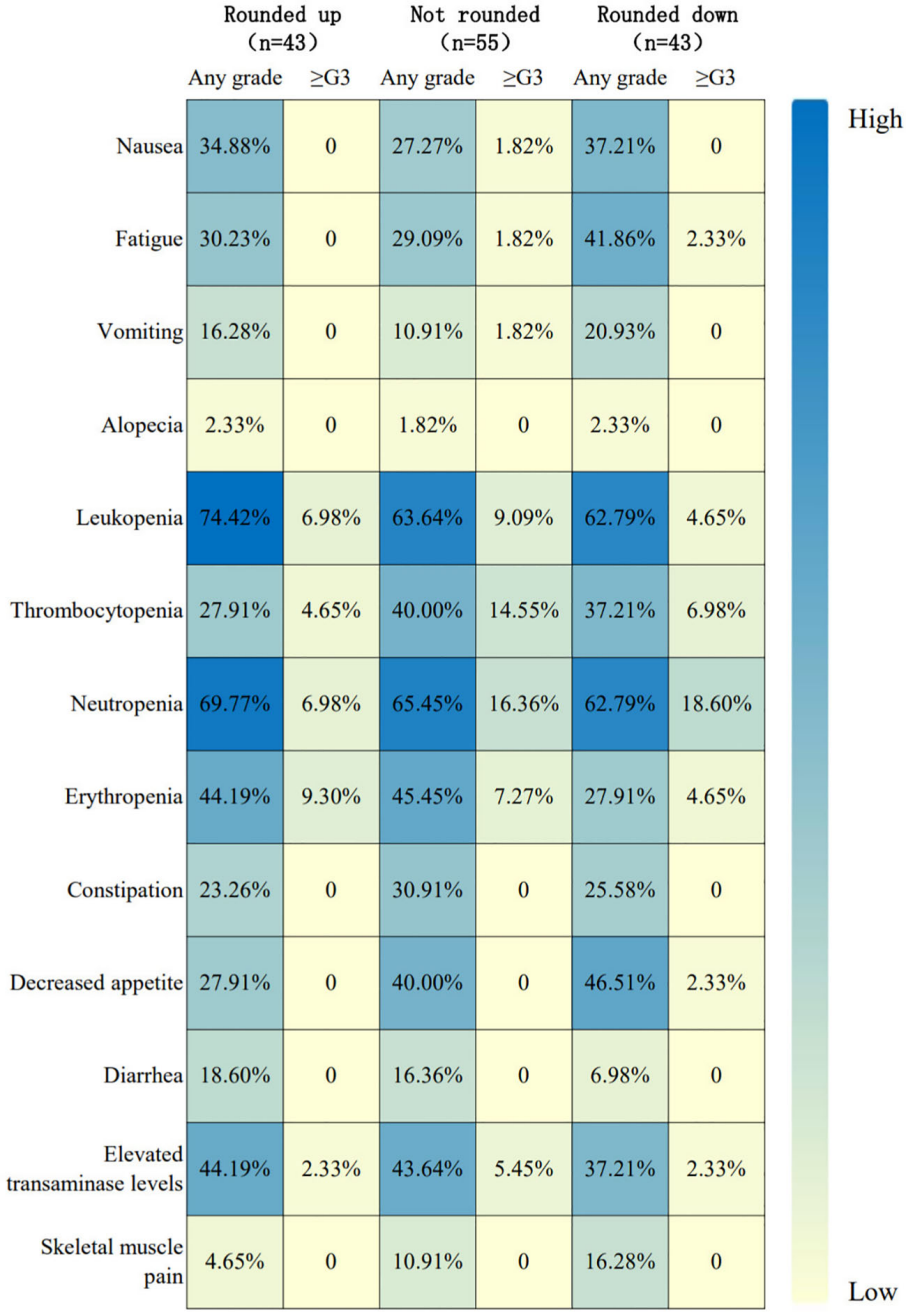
Adverse reaction comparison heatmap. Data within cells are expressed as “incidence rate (%)”, and the intensity of color indicates the severity of adverse reaction incidence.

The incidence of adverse reactions among patients in the dose rounding up, no dose rounding, and dose rounding down groups is presented in **Figure 2**.

The color distribution patterns for adverse reactions of any grade and ≥Grade 3 were highly consistent across the three patient groups, with no significant intergroup differences observed. Hematologic toxicity was the predominant adverse event type, with incidence rates exceeding 55% for leukopenia, neutropenia, and anemia. However, Grade 3–4 events were less frequent, occurring at rates of 4.65%-9.09%, 6.98%-18.60%, and 4.65%-9.30%, respectively, with no significant intergroup differences. Non-hematologic adverse reactions such as fatigue, decreased appetite, and elevated transaminase levels occurred in all three groups, predominantly Grade 1-2, with few ≥Grade 3 events. These findings did not compromise the overall safety profile of treatment. Given the emetogenic potential of trastuzumab deruxtecan, most patients received 2 or 3 prophylactic agents (e.g., dexamethasone plus a 5-HT3 receptor antagonist and/or an NK1 receptor antagonist) before each cycle to prevent chemotherapy-induced nausea and vomiting. Consequently, the incidence of nausea and vomiting was lower than that reported in clinical trials. Additionally, adverse reactions such as alopecia, constipation, diarrhea, and skeletal muscle pain occurred at relatively low rates, with no Grade 3 or 4 events reported.

Chi-square tests (cross-tabulation analysis) were performed to compare the incidence of adverse events among the three groups (dose rounding up, no dose rounding, and dose rounding down). Results are presented in **Table 3**. No significant differences were observed in the incidence of any-grade adverse events, including hematologic and non-hematologic toxicities, among the three groups (P>0.05).

**Table 3:** Multiple comparisons of adverse drug reactions (chi-square test).

Type of ADR	Any grade					≥G3				
	Rounded up (cases)	Not rounded (cases)	Rounded down (cases)	x2	P	Rounded up (cases)	Not rounded (cases)	Rounded down (cases)	x2	P
Nausea	15	15	16	1.228^a^	0.541	0	1	0	1.575^a^	0.455
Fatigue	13	16	18	2.038^a^	0.361	0	1	1	0.935^a^	0.627
Vomiting	7	6	9	1.862^a^	0.394	0	1	0	1.575^a^	0.455
Alopecia	1	1	1	0.041^a^	0.979	0	0	0	0.000^a^	1
Leukopenia	32	35	27	1.681^a^	0.432	3	5	2	0.723^a^	0.697
Thrombocyto-penia	12	22	16	1.625^a^	0.444	2	8	3	3.195^a^	0.202
Neutropenia	30	36	27	0.476^a^	0.788	3	9	8	2.740^a^	0.254
Erythropenia	19	25	12	3.620^a^	0.164	4	4	2	0.710^a^	0.701
Constipation	10	17	11	0.777^a^	0.678	0	0	0	0.000^a^	1
Decreased appetite	12	22	20	3.260^a^	0.196	0	0	1	2.295^a^	0.317
Diarrhea	8	9	3	2.740^a^	0.254	0	0	0	0.000^a^	1
Elevated transaminase levels	19	24	16	0.549^a^	0.76	1	3	1	0.960^a^	0.619
Skeletal muscle pain	2	6	7	3.065^a^	0.216	0	0	0	0.000^a^	1

Chi-square tests were similarly used to evaluate differences in the occurrence of adverse events of Grade 3 or higher among the different dose-rounding groups. The occurrence of Grade 3–4 adverse events, including hematologic and non-hematologic toxicities, showed no statistically significant differences between the dose rounding up, no dose rounding, and dose rounding down groups (P>0.05).

## 4 Discussion

The global number of patients with cancer continues to increase, and exorbitant drug prices impose a substantial financial burden on patients and their families. Growing evidence suggests that dose rounding strategies for anticancer biologics can effectively reduce drug wastage and lower treatment costs while preserving therapeutic efficacy. This approach involves adjusting the actual administered dose to the nearest available vial size. Accordingly, this study analyzed the clinical safety and cost-effectiveness of dose rounding for trastuzumab deruxtecan based on hospital HIS data and relevant international initiatives.

### 4.1 Clinical safety

The results of this study indicate that the incidence of any-grade and ≥Grade 3 hematologic adverse reactions (leukopenia, neutropenia, anemia, thrombocytopenia) was comparable among the dose rounding-up, dose rounding-down, and non-rounding groups. No statistically significant differences were observed between groups. Non-hematologic adverse events were predominantly Grade 1-2, with no significant differences in incidence or severity across groups. No additional safety risks attributable to dose rounding were identified in the three cohorts, and no new or unexpected adverse reactions were detected. These findings are consistent with the safety profile described in the trastuzumab deruxtecan prescribing information (13). They also align with the conclusions of Moore DC et al. (14), who rounded bevacizumab doses to the nearest 100 mg or 400 mg vial size. Their study showed no significant differences in adverse reaction rates (e.g., hypertension, proteinuria) between patients receiving upward-rounded and downward-rounded doses, suggesting that dose rounding up may be acceptable under strict ±10% limits, but should not be routinely promoted without further evidence to maintain medication safety and treatment efficacy. Furthermore, international initiatives, including the *Global initiative to establish and implement dose rounding policy for expensive cancer therapy (*4) and related foreign studies (9), have confirmed that standardized dose rounding of antineoplastic biologics can effectively reduce drug wastage and lower medical costs without compromising treatment safety. The study focused on ADC dose rounding in a real-world Chinese population, validating the safety and feasibility of international dose rounding strategies in this population and demonstrating its broad application potential.

Unlike traditional cytotoxic drugs, the dosage of trastuzumab deruxtecan is based on target saturation rather than the maximum tolerated dose (15), resulting in a wide therapeutic window. Because theoretical doses calculated based on body surface area or body weight inherently show interindividual variability, minor dose rounding within a reasonable range (fluctuation ≤ 10%) leads to dose changes far smaller than the natural pharmacokinetic variability among individual patients. Such adjustments generally do not significantly alter systemic drug exposure or increase the risk of adverse events. In addition, the incidence and severity of trastuzumab deruxtecan related adverse events are more dependent on patient specific factors such as organ function, age, and prior treatment history (16). Dose variations within 10% are unlikely to have a significant impact on these outcomes.

The baseline characteristics of the three patient groups were comparable, with no significant differences in age, weight, physical condition, underlying diseases, or concomitant medications. This minimized the influence of baseline confounding factors on safety outcomes. Although rounding doses to the nearest 100 mg may lead to fluctuations exceeding 10% in some patients, especially those with lower body weight, all rounded doses in this clinical practice remained within 10% variation and fully met internationally recommended standards.

Prophylactic supportive care may have influenced the observed adverse event rates. In this study, most patients received standardized antiemetic prophylaxis (dexamethasone plus 5-HT3 antagonist, with or without NK1 antagonist) prior to each cycle. However, the use of granulocyte colony-stimulating factors (G-CSF) and other supportive measures was comparable across the three groups (data not shown), suggesting that differential supportive care did not account for the lack of increased toxicity in the rounded-up group.

Although body weight differed significantly between the rounded-up and non-rounded groups (60.4 kg vs. 53.7 kg, P = 0.0003), this difference did not translate into a lower adverse event rate in the rounded-up group. The observed dose increases (within 10%) corresponded to small absolute increments (0.4-27.2 mg), which are unlikely to meaningfully alter systemic exposure given the wide therapeutic window of trastuzumab deruxtecan.

### 4.2 Economic outcomes

This study collected data from patients treated with trastuzumab deruxtecan over a two-year period at a tertiary cancer hospital. A total of 829 administrations were included, involving 514 instances dose rounding, including dose rounding up and dose rounding down. This resulted in a total treatment cost savings of CNY 897, 840, while avoiding 4, 368.3 mg of trastuzumab deruxtecan wastage. Field K et al. (17) analyzed oxaliplatin usage from prospective databases of four hospitals. They calculated a potential cost savings of approximately AUD 51–898 over 5 years for patients with BSA between 1.77 m^2^ and 1.94 m^2^. Extrapolated to the Australian population, this could save more than AUD 2.5 million per year. Monoclonal antibody agents are associated with high unit prices and are widely used in clinical practice. Compared with chemotherapy drugs, dose rounding for monoclonal antibody agents accounted for only 15.98% of administrations but contributed 45.27% of total cost savings (10). This indicates that widespread implementation of dose rounding strategies for ADC drugs such as trastuzumab deruxtecan will have substantial economic value.

Park JJ et al. (18) conducted a retrospective review of electronic medical records and found that standardized dose rounding for infliximab was associated with lower drug costs compared with non-standardized rounding. After excluding the initial period when the standardized dosing table was first implemented, the study also showed significantly shorter prescription review times and improved pharmacy workflow efficiency. Our hospital has also implemented a standardized dose rounding, with unified processes for order writing, dose calculation, and pharmacist review. This approach has reduced repetitive checks and dose adjustments related to non-standardized doses, effectively improved clinical efficiency, and lowered the risk of human calculation errors. The strategy achieves both economic benefits and clinical convenience, and provides technical support for drug cost control and waste reduction in medical institutions in China. It should be noted that dose rounding up does not reduce the number of vials dispensed compared with the prescribed dose; rather, it utilizes the residual volume that would otherwise be discarded. Therefore, the financial benefit is limited to waste reduction, not direct cost savings for payers or patients.

### 4.3 Limitations

Trastuzumab deruxtecan is a single-dose preparation without preservatives. When dose rounding is not performed, the theoretical dose calculated based on body weight often results in wastage of the remaining solution after vial opening, as it cannot be stored. For drugs such as trastuzumab (Herceptin) that are available in multi-dose formulations or contain preservatives, the unused portion can be preserved. Therefore, the cost-effectiveness of dose rounding may be reduced for these agents.

Limitations of this study also include its small sample size, retrospective design and single center setting. The limited sample size and short follow up duration make it difficult to fully evaluate the impact of dose rounding on long term efficacy outcomes such as progression free survival and overall survival. However, the aim of this study was to investigate the safety and economic outcomes in the early period after initial implementation of the dose rounding policy. The present findings have confirmed its clinical value and demonstrated that international dose rounding strategies can also be applied to patients in the Chinese population. Future multi-center, prospective studies with larger sample sizes and extended follow-up are needed to evaluate the impact of dose rounding on long-term efficacy endpoints, including progression-free survival (PFS) and overall survival (OS), as well as to confirm the safety findings in diverse populations. Alternatively, fixed dosing within predefined body weight ranges could eliminate the need for per-administration rounding and further simplify clinical workflows. This approach warrants investigation in future studies.

Taken together, the study investigated clinical dose rounding strategies for trastuzumab deruxtecan based on foreign guidelines and research. The results showed that dose rounding down can reduce drug costs, while dose rounding up within ±10% can minimize drug waste, without significantly affecting the incidence or severity of adverse events.This study confirms that international dose rounding strategies are also suitable for clinical practice in China. It provides a local reference for the rational use and cost control of domestic oncology drugs, and further strengthens the theoretical understanding of the appropriate use of new antineoplastic agents.

## Data Availability

The data analyzed in this study is subject to the following licenses/restrictions: The raw datasets used in this study contain protected health information (PHI) of patients. Due to patient privacy protection laws and institutional data governance policies, the datasets cannot be made publicly available or included in the article/supplementary materials. De-identified, aggregated data may be made available from the corresponding author upon reasonable request and with approval from the institutional review board. Requests to access these datasets should be directed to Haochun Tang, 706370277@qq.com.
